# A neuroscientific account of how vestibular disorders impair bodily self-consciousness

**DOI:** 10.3389/fnint.2013.00091

**Published:** 2013-12-06

**Authors:** Christophe Lopez

**Affiliations:** Laboratoire de Neurosciences Intégratives et Adaptatives - UMR 7260, Centre Saint Charles, Fédération de Recherche 3C, Centre National de la Recherche Scientifique - Aix-Marseille UniversitéMarseille, France

**Keywords:** vestibular system, body schema, body image, touch, caloric vestibular stimulation, bodily consciousness, multisensory integration

## Abstract

The consequences of vestibular disorders on balance, oculomotor control, and self-motion perception have been extensively described in humans and animals. More recently, vestibular disorders have been related to cognitive deficits in spatial navigation and memory tasks. Less frequently, abnormal bodily perceptions have been described in patients with vestibular disorders. Altered forms of bodily self-consciousness include distorted body image and body schema, disembodied self-location (out-of-body experience), altered sense of agency, as well as more complex experiences of dissociation and detachment from the self (depersonalization). In this article, I suggest that vestibular disorders create sensory conflict or mismatch in multisensory brain regions, producing perceptual incoherence and abnormal body and self perceptions. This hypothesis is based on recent functional mapping of the human vestibular cortex, showing vestibular projections to the primary and secondary somatosensory cortex and in several multisensory areas found to be crucial for bodily self-consciousness.

## INTRODUCTION

The consequences of vestibular disorders are dramatic as they incorporate a wide range of symptoms including vertigo, loss of balance, and blurred vision. It is accepted that vertigo results from the activation of the vestibulo-thalamo-cortical pathways, postural instability and falls from abnormal vestibulo-spinal reflexes, and blurred vision from impaired vestibulo-ocular reflexes (Curthoys and Halmagyi, 1995; Borel et al., 2008). More recently, deficits in spatial navigation and memory tasks have been related to vestibular disorders, presumably due to vestibular projections to the cortex and hippocampus (Smith, 1997; Brandt et al., 2005).

In addition to these deficits, vestibular patients sometimes report abnormal bodily perceptions. The role of vestibular organs in bodily perceptions has captured the attention of pioneering researches on body representations such as those of Bonnier (1893, 1905), Schilder (1935); Lhermitte (1939), and Menninger-Lerchenthal (1946). These authors reported cases of patients losing connections with their body, experiencing deformations of their body, or disembodiment. Yet, the mechanisms underpinning these disorders remain poorly understood. One reason is that bodily disorders have to date not been quantified experimentally in vestibular patients despite the development of psychophysical methods to measure various bodily experiences (Blanke, 2012). Secondly, the comprehension of how vestibular dysfunction modifies bodily consciousness has been hampered by scarce descriptions of the vestibular cortex. In the present article, I argue that recent progresses in functional mapping of the human vestibular cortex and advances in the neuroscience of bodily self-consciousness afford a neuroscientific explanation of the mechanisms at the basis of bodily disorders in vestibular patients.

## A NEUROSCIENTIFIC FRAMEWORK BASED ON THE MULTISENSORY NATURE OF THE VESTIBULO-THALAMO-CORTICAL PATHWAYS

**FIGURE 1:**
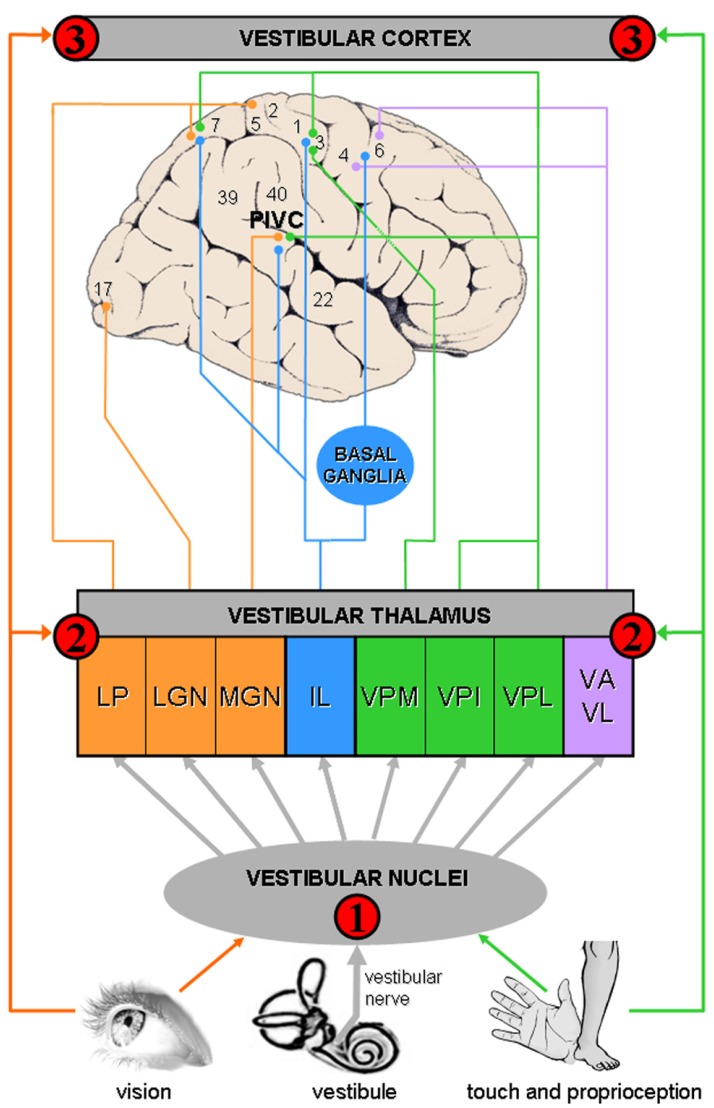
**Convergence of vestibular, visual, and somatosensory signals in vestibulo-thalamo-cortical structures.** The schema summarizes animal and human data, showing multisensory convergence in three vestibular relays (see also **Table 1** for details):
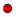
Vestibular signals are first processed in the *vestibular nuclei* in the brainstem, a region that is highly multisensory. 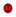
A second level of vestibular processing takes place in the *thalamus*. Multiple thalamic nuclei contain neurons that respond to vestibular stimulation such as the ventroposterior complex (VPM, ventral posterior medial nucleus; VPI, ventral posterior inferior nucleus; VPL, ventral posterior lateral nucleus), ventroanterior (VA) and ventrolateral (VL) nuclear complex, intralaminar nuclei (IL), thalamic posterior nuclear group (MGN, medial geniculate nucleus; LGN, lateral geniculate nucleus) and lateral posterior nucleus (LP). Most of these thalamic nuclei contain multisensory neurons. 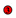
A third level of vestibular processing occurs in the *cerebral cortex*. Neuroimaging studies used caloric (CVS) and galvanic (GVS) vestibular stimulation and revealed activations centered on the insula, parietal operculum, and temporo-parietal junction (Lopez et al., 2012a; zu Eulenburg et al., 2012). This area may be similar to a region known as the *parieto-insular vestibular cortex* (PIVC) in monkeys (Grüsser et al., 1990a,b; Guldin and Grüsser, 1998; Chen et al., 2010). The PIVC is considered the core region of the vestibular cortex because it is strongly connected or interconnected with most of the other vestibular cortical areas. At least 10 other cortical areas process vestibular signals including somatosensory (areas 2 and 3), superior parietal, cingulate, and premotor cortex.

The neuroscientific framework to understand bodily disorders in vestibular patients is based on the multisensory nature of the vestibulo-thalamo-cortical pathways, a finding that was unknown from Bonnier and Schilder when they described the consequences of vertigo on body perception. A vestibulo-visuo-somatosensory convergence has been found in all vestibular relays, including vestibular nuclei, thalamus, and cerebral cortex (see **Figure 1** and **Table 1** for details).

**Table 1:** Evidence of multisensory integration in three vestibulo-thalamo-cortical structures.

Anatomical structure	Evidence of multisensory integration
Vestibular nuclei	Vestibular nuclei neurons respond to vestibular, visual (optokinetic stimulation: Waespe and Henn, 1978), tactile, proprioceptive (Roy and Cullen, 2004) and eye movements signals (Tomlinson and Robinson, 1984). These neurons integrate signals from otoliths and semicircular canals afferents to discriminate between head translations and head tilts, as well as to distinguish between active (or voluntary) and passive (or involuntary) head movements (review in Angelaki and Cullen, 2008).
Thalamus	Vestibular thalamic neurons are characterized by very similar responses that have been described for vestibular nuclei neurons, i.e., they respond to visual, tactile, and proprioceptive stimuli (review in Lopez and Blanke, 2011). For example, about half of the vestibular neurons in the cat LGN respond to optokinetic stimulation (Magnin and Putkonen, 1978) and most of them are also driven by saccadic eye movements. Vestibular neurons in the VPL, VPM, and VPI respond to proprioceptive signals from joints and muscles (Deecke et al., 1977; Blum and Gilman, 1979) and code for passive movements of the neck, shoulders, legs, and vertebral column. Thalamic vestibular neurons also respond to tactile stimulation applied to the animal’s paws (Sans et al., 1970).
Cerebral cortex	Vestibulo-visuo-somatosensory convergence has been reported in the PIVC, at the junction of the insula with the retroinsular and somatosensory cortex (Grüsser et al., 1990a,b; Guldin and Grüsser, 1998). Visual–vestibular convergence has also been reported in the extrastriate visual area MST, a major region for self-motion perception based on optic flows (Bremmer et al., 1999; Gu et al., 2006). Vestibular–somatosensory convergence has been reported in the intraparietal sulcus and primary somatosensory cortex in monkeys (areas 2v and 3av in the hand/arm and neck/trunk representations) and in the secondary somatosensory cortex in humans (Schwarz and Fredrickson, 1971; Bottini et al., 1995, 2001, 2005; Guldin and Grüsser, 1998; Bremmer et al., 2002; Fasold et al., 2008).

Normal sensorimotor development calibrates synergies between actions and their sensory consequences at both behavioral and neural levels (Held and Hein, 1963). For example, head rotations to the right are normally encoded with leftward optic flow and matching proprioceptive signals from the neck. Corresponding synergistic responses exist in all vestibulo-thalamo-cortical structures and recent studies showed that vestibular and visual responses combine “in a statistically optimal fashion,” in accordance with the predictions of Bayesian models (MacNeilage et al., 2007; Fetsch et al., 2012). Importantly, sensory conflicts may disorganize calibrated synergies at the neural level (e.g., visuo-vestibular mismatch alters neural responses in vestibular nuclei; Waespe and Henn, 1978). Here, I propose similar mismatch is produced by various peripheral vestibular disorders (e.g., Menière’s disease, vestibular neuritis). I suggest that vestibular disorders provide the brain with erroneous vestibular signals about current self-motion and position, and create *sensory conflicts* (or *mismatch*) leading to a *perceptual incoherence*. That is, abnormal vestibular signals would induce misinterpretation of tactile, proprioceptive and visual signals from the body and, as a consequence, distort bodily self-experience. In support of this view are recent data on self-motion perception showing that even in the case of large visual–vestibular conflicts, vestibular information is not disregarded and both signals are “mandatorily fused” (Prsa et al., 2012). Other studies suggest that during multisensory conflicts, vestibular cues are weighted higher (Butler et al., 2010; Fetsch et al., 2012). These data indicate that participants strongly rely on vestibular signals, even when this information contradicts other sensory cues.

In the following sections, I describe a detailed neuroscientific account of how vestibular dysfunction can distort various aspects of the bodily self.

## DISTORTED BODY SCHEMA AND BODY IMAGE

### CLINICAL DESCRIPTION

Vestibular disorders may impair two fundamental aspects of mental body representations known as *body schema* and *body image*. They refer to different types of representations of body configuration and metric properties, including the size and shape of body parts, and body position in space (e.g., Gallagher, 2005; Berlucchi and Aglioti, 2010; de Vignemont, 2010; Longo et al., 2010; Serino and Haggard, 2010). Although body schema and body image have been proposed to be of mostly proprioceptive and visual origin, a vestibular contribution was postulated over a century ago. Bonnier (1893, 1905) described striking examples of distorted perceptions of the body shape and size in vestibular patients. For example, one of his patients “felt his head became enormous, immense, losing itself in the air; his body disappeared and his whole being was reduced to only his face.” Interestingly, Bonnier coined the term “*aschématie*” (indicating a “loss” of the *schema*) to describe distorted representations of the volume, shape, and position of the body and body segments (Vallar and Papagno, 2003; Vallar and Rode, 2009). Several decades later, Schilder (1935) described distorted body schema and image in vestibular patients who reported that the “neck swells during dizziness,” “extremities had become larger,” or “feet seem to elongate” (p. 117). Altogether, these sensations are comparable to neurological symptoms of *asomatognosia* (e.g., Dieguez et al., 2007), even if evoked solely by peripheral vestibular disorders.

### EXPERIMENTAL EVIDENCE

Several lines of evidence from neurology and experimental psychology support the idea that abnormal body image and schema might change due to misinterpretation of bodily signals created by vestibular disorders. All of them are based on studies showing the influence of caloric (CVS) and galvanic (GVS) vestibular stimulation on the perceived shape and size of the body. Rode et al. (2012) described a patient with Wallenberg’s syndrome who reported a macrosomatognosia restricted to his left hemiface. In this patient, CVS temporarily alleviated distorted face perception. CVS also changed the perceived shape and position of phantom limbs in paraplegics (Le Chapelain et al., 2001). Similarly, CVS evoked the perception of a phantom limb in amputees who did not experience phantoms before, or altered the phantom perception in those who experienced phantoms already (André et al., 2001). This indicates that CVS can influence mental representations of a no-longer existing body segment and suggests that vestibular signals project to multisensory brain regions representing the body’s metric properties. Yet, these observations were based only on patients’ reports. Lopez et al. (2012b) demonstrated similar influence of CVS by using psychophysical measures in healthy participants. The influence of CVS on the perceived shape and size of the body was measured using a tactile distance comparison task and a proprioceptive judgment task. The results showed that CVS known to stimulate the right cerebral hemisphere modified the perceived size of the left hand, that appeared to be enlarged. This finding was later corroborated by the application of GVS (Ferrè et al., 2013).

### NEUROPHYSIOLOGICAL HYPOTHESIS

Neuroimaging studies have revealed the implication of the posterior parietal cortex in body shape and size perception. In particular, the perception of the current position of body segments is thought to rely on the superior parietal lobule and intraparietal region (Wolpert et al., 1998; Félician et al., 2004; Corradi-Dell’Acqua et al., 2008, 2009). The inferior parietal lobule is also particularly relevant since electrical stimulation of the angular gyrus distorted body schema in epileptic patients (Blanke et al., 2002) and because transcranial direct current stimulation applied over the right angular gyrus modified body representations (Spitoni et al., 2013). Neuroimaging studies further revealed the implication of the parietal operculum and posterior insula as they contain somatotopic representations of the body (Eickhoff et al., 2007; Corradi-Dell’Acqua et al., 2009; Hashimoto and Iriki, 2013). Importantly, these parietal and insular regions process vestibular signals and the parietal operculum has even been proposed as the core vestibular cortex (Guldin and Grüsser, 1998; Eickhoff et al., 2006; Lopez et al., 2012a; zu Eulenburg et al., 2012). It is interesting to note that animal data revealed vestibulo-somatosensory convergence in parietal cortex, intraparietal sulcus, and operculo-insular cortex (Grüsser et al., 1990a,b, 1994; Bremmer et al., 2002). Bottini and colleagues showed that the parieto-insular cortex is a region where CVS interferes with tactile perception (Bottini et al., 1995, 2001, 2005; Ferrè et al., 2012). Accordingly, abnormal vestibular signals arriving in these regions during vertigo attacks may interfere with somatosensory processing. The misinterpretation of postural somesthetic signals from the neck may explain the patients’ reports that their neck or head is enlarged. In support of this view is the fact that CVS and GVS produce similar effects in healthy volunteers (Lopez et al., 2012b; Ferrè et al., 2013).

## EMBODIMENT OR THE SENSE OF UNITY BETWEEN THE SELF AND THE BODY

### CLINICAL DESCRIPTION

Vestibular patients may lose connection with their body and may be subject to an out-of-body experience (OBE). During an OBE, subjects localize their self outside their body, at a location that is often elevated (i.e., floating in the room), and experience seeing the environment from this disembodied location. Subjects may also experience seeing their own body (i.e., autoscopy), a double with which they strongly self-identify (Brugger, 1997; Blanke et al., 2004; Blanke and Mohr, 2005; Lopez et al., 2008; Blanke, 2012). Yet, clear cases of full-blown OBEs due to vestibular disorders seem very rare. Bonnier (1905) described the case of a loss of self–body unity: “it seemed to [the patient] that he was divided into two persons, one who had not changed posture, and another new person on his right, looking somewhat outwardly. Then the two somatic individuals approached each other, merged, and the vertigo disappeared.” Illusory perceptions of doubles in vestibular pathology were also reported by Skworzoff (1931): one patient saw herself (i.e. autoscopy) for a moment in day light (Case 5, p. 764). Another patient saw and felt every day his own double (Case 6, p. 764). The same patient also reported in some instances sensations of flying, which could be evocative of an otolithic dysfunction.

### EXPERIMENTAL EVIDENCE

Vestibular stimulation in healthy participants can strongly modify experienced self-location. GVS creates illusory motion of the entire body, i.e., dissociation between the perceived self-location (that appears tilted toward the cathode) and physical body location (Fitzpatrick and Day, 2004; Lenggenhager et al., 2008). Lopez et al. (2008) have proposed that such dissociation between self and body location reflects a type of *partial disembodiment* that is reminiscent of OBEs of neurological origin. Another indirect evidence of a vestibular contribution to embodied self-location comes from the observation that OBEs are more frequent in patients lying than sitting or standing upright (Blanke and Mohr, 2005). According to Green (1968), about 73% of OBEs occur spontaneously when healthy subjects are lying down, and less often in sitting or standing subjects, suggesting a strong contribution of gravitational vestibular signals to self-location and embodiment.

### NEUROPHYSIOLOGICAL HYPOTHESIS

A neurophysiological model by Blanke and colleagues posits that during OBEs a *triple sensory misintegration*, with conflicting vestibular, visual, and somatosensory signals, may occur in multisensory brain regions such as the temporo-parietal junction (Blanke et al., 2002, 2004; Blanke and Mohr, 2005; Lopez et al., 2008; Blanke, 2012). This model is supported by the fact that vestibular sensations (e.g., floating, lightness, elevation) occur often during OBEs of neurological origin (Devinsky et al., 1989; Blanke et al., 2002, 2004; Lopez et al., 2010; Heydrich et al., 2011). In addition, brain areas that are the most commonly damaged in OBE patients overlap with the vestibular cortex at the temporo-parietal junction (Blanke et al., 2004; Ionta et al., 2011). Altogether, these data suggest a close relation between the phenomenal experience of a disembodied self and vestibular misintegration (Lopez and Blanke, 2007; Lopez et al., 2008; Blanke, 2012). Accordingly, the loss of self–body unity in vestibular patients may be due to sensory mismatch created by vertigo attacks at the temporo-parietal junction and posterior insula, two regions the metabolism of which is strongly disorganized by vestibular disorders (Bense et al., 2004; Alessandrini et al., 2013). Interfering with the temporo-parietal junction by electrical stimulation has also been showed to induce both OBE and vestibular sensations (Penfield, 1955; Blanke et al., 2002; De Ridder et al., 2007). Vertigo attacks may produce a similar type of interference as those intracranial stimulations, but to a weaker extent, since full-blown OBEs were rarely reported in vestibular pathology.

## AGENCY

### CLINICAL DESCRIPTION

The loss of self–body connection described above is also evident in the motor control domain. A minimal sense of selfhood has been related to the *sense of agency*, the experience of being the agent of one’s own actions (Franck et al., 2001; Jeannerod, 2006, 2009). Interestingly, vestibular patients report more often than healthy participants the experience of “not being in control of their self” (Sang et al., 2006; Jauregui-Renaud et al., 2008b). For example, a patient with a bilateral Menière’s disease reported during vertigo attacks “watching something happen and not being a part of it. It’s just a feeling of not being there, participating in what’s going on” (Case 2, p. 532 in Grigsby and Johnston, 1989). Vestibular patients often report that their actions do not seem to match their intentions. Even when tested at a compensated stage of a vestibular loss, patients perceive instability and dizziness during walking and standing despite no evident sign of postural unbalance.

### EXPERIMENTAL EVIDENCE

To date, the role of vestibular signals in the sense of agency has not been measured experimentally. However, it has been showed that CVS evoked in healthy participants significantly stronger feeling of “not being in control of the self” than control stimulation (Lopez et al., 2012b). In addition, GVS altered the ability to perform and predict hand movements (Bresciani et al., 2002; Guillaud et al., 2011), but agency was not measured directly in these experiments.

### NEUROPHYSIOLOGICAL HYPOTHESIS

Vestibular patients may report altered sense of agency because vestibular organs do not correctly encode the consequences of their actions. Patients tend to underestimate their body displacements and misinterpret the direction of body movements (Cohen, 2000; Borel et al., 2004), revealing the crucial role of vestibular signals in spatial updating during active and passive whole-body motions (e.g., Frissen et al., 2011; Campos et al., 2012). Errors in sensory coding can introduce a mismatch between vestibular, visual, and somatosensory feedback about self-initiated movements, as well as a discrepancy with the efferent signals from the motor command. Behavioral studies showed that agency is based on congruent sensory feedback from one’s actions: introducing a mismatch (amplitude or direction of motion) between the visual and proprioceptive consequences of an action impairs agency (Fourneret and Jeannerod, 1998; Farrer et al., 2003b, 2008a; Kannape et al., 2010). I speculate that an additional factor may be responsible for distorted sense of agency in vestibular patients: a *temporal mismatch* between an action and the sensory feedback from this action. Interestingly, perception of time is altered in vestibular patients (Israel et al., 2004) and introducing a delay between the executed and seen movement disturb agency (Franck et al., 2001). Neuroimaging studies have revealed that the insula and the temporo-parietal junction are involved in agency (Spence et al., 1997; Farrer and Frith, 2002; Farrer et al., 2003a, 2004, 2008b). This reiterates the contribution of two main vestibular regions to a crucial bodily experience of self-consciousness. In light of these points, vestibular dysfunctions are therefore likely to create a spatiotemporal mismatch between efferent motor commands and feedback from an action, resulting in a disturbed sense of agency.

## DEPERSONALIZATION AND DEREALIZATION

### CLINICAL DESCRIPTION

Most of the bodily disturbances described in the previous sections are part of depersonalization, a dissociative disorder characterized by the loss of familiarity of the self and surrounding and by a detachment from the self, that may be experienced as unreal (Simeon and Abugel, 2006). Early in the last century, Schilder (1914, 1935) already proposed a vestibular contribution to depersonalization and derealization (DD). More recently, Grigsby and Johnston (1989) collected experiences of DD in Menière’s disease patients: a patient described DD as “a sense of unreality” and claimed “I feel like I’m outside of myself. I feel like I’m not in myself” (p. 531). Another patient reported “I am not actually being there or having anything to do with my body” (p. 532). More recently, the use of the Cox and Swinson (2002) questionnaire revealed that vestibular patients have more frequent and more severe DD symptoms than controls (Sang et al., 2006; Jauregui-Renaud et al., 2008b). Symptoms included sensations of “déjà vu,” “body feels strange” and the experience of feeling “spacey” or “spaced out.”

### EXPERIMENTAL EVIDENCE

Yen Pik Sang et al. (2006) applied CVS in healthy volunteers and showed that it increased the frequency of DD symptoms such as “surroundings seem strange and unreal,” “time seems to pass very slowly” and “body feels strange/different in some way.” This finding was confirmed during bilateral CVS (Lopez et al., 2012b). It is not known, however, how long these effects persist.

### NEUROPHYSIOLOGICAL HYPOTHESIS

Discrepancy between vestibular and other body-related signals may deteriorate the experience of the body and surroundings, leading to DD. In line with this view is the observation that various sensory dysfunctions increase the frequency of DD symptoms (vestibular, visual, auditory: Jauregui-Renaud et al., 2008a,b; somatosensory: Lenggenhager et al., 2012). The superior temporal and inferior parietal cortices are the best candidates to explain the vestibular influence on DD. During stimulation of the superior temporal cortex, Penfield (1947, 1955) evoked sensations of “déjà vu” and altered self-body relations (“I feel queer, as though I were floating away” and “I have a queer sensation as if I am not here,” Penfield, 1947, p. 342). Interestingly, the sites of these stimulations overlapped those where vestibular sensations were evoked. In a PET study, Simeon et al. (2000) showed that DD were related to changes in brain metabolisms in regions that were also activated by CVS and GVS (superior temporal gyrus and temporo-parietal junction). Of particular interest here is the study by Bense et al. (2004) showing that very similar regions have altered metabolism during vestibular neuritis. This anatomical overlap strongly suggests that vestibular dysfunction disorganizes brain metabolism, multisensory integration, and eventually structure and connections in the multisensory temporo-parietal cortex, and this may be the underlying mechanism of DD in vestibular patients.

## CONCLUSION

A better understanding of cortical vestibular processing, as well as of how CVS and GVS influence body and self perceptions, has provided the basis for a neuroscientific account of a so far under-recognized type of vestibular symptom – alterations in bodily self-consciousness. I have summarized evidence showing that abnormal forms of bodily self-consciousness in vestibular disorders may result from sensory conflict or mismatch in multisensory brain regions. This hypothesis should now be put under scientific scrutiny by correlating changes in bodily self-consciousness (e.g., subjective reports using DD questionnaires and objective measures of altered sense of agency and self-location; Kannape et al., 2010) with changes in metabolism and structure in the vestibular cortex (e.g., Bense et al., 2004; voxel-based morphometry: zu Eulenburg et al., 2010). In addition, I have drawn parallels between experimental evidence and clinical observations in vestibular patients to clarify the neural and sensory mechanisms of bodily self-consciousness. Future research in the field should endeavor to make the same comparisons to further our understanding of the underlying multisensory mechanisms of bodily self-consciousness (see Blanke, 2012). In particular, several bodily experiences should now be systematically quantified in vestibular patients to obtain a full description of the consequences of vestibular dysfunctions, including changes in the patient’s *self*, mood, and personality. I am optimistic that these data will also impact on the multisensory models of self-consciousness currently developed by neuroscientists and philosophers and in which the contribution of the vestibular system is often neglected.

## Conflict of Interest Statement

The author declares that the research was conducted in the absence of any commercial or financial relationships that could be construed as a potential conflict of interest.
